# Causal Associations Between Major Depression and Accelerated Biological Aging.

**DOI:** 10.1093/geroni/igaf122.2083

**Published:** 2025-12-31

**Authors:** Breno Diniz, Shangshu Zhao, Richard Fortinsky, George Kuchel, Chia-Ling Kuo

**Affiliations:** UConn Health, Farmington, Connecticut, United States; UConn Health, Farmington, Connecticut, United States; University of Connecticut, Farmington, Connecticut, United States; UConn Health, Farmington, Connecticut, United States; UConn Health, Farmington, Connecticut, United States

## Abstract

Over the past decades, there is growing evidence from clinical and epidemiological studies that history of major depression is associated with adverse health outcomes, including higher incidence of Alzheimer’s disease, cardiovascular diseases, and mortality. The mechanisms for such associations are unclear, but points to the fact that major depression is associated with abnormalities in multiple hallmarks of biological aging. Therefore, this analysis aimed to investigate if major depression was causally linked with accelerated biological aging, based on proteomic measures. We carried out a bidirectional Mendelian randomization analysis to evaluate the causal relationships between these two phenomena. We focused on 3 proteomic measures of biological aging acceleration: The Proteomic Aging Clock (PAC), the Healthspan Proteomic Score (HPS), and the Brain-specific Proteomic Aging (BsPA). We found that MDD was causally associated with biological aging acceleration (Major Depression Biological Aging) for all 3 measures (PAC: MR-IVW β = 0.07, p = 0.02; HPS: MR-IVW β=-0.02, p < 0.001; BsPA: MR-IVW β = 0.09, p = 0.002). However, we found no statistical evidence that biological aging was causally linked with major depression (Biological Aging Major Depression). Our results provides robust evidence that genetically determined risk of major depression is causally associated with accelerated biological aging and provides a mechanistic perspective for the worse adverse health outcomes observed in these individuals. Also, it highlights the importance of prevention and treatment of major depression across the lifespan to mitigate the speed of biological aging acceleration.

